# A Comparison of Conventional Collagen Sponge and Collagen-Gelatin Sponge in Wound Healing

**DOI:** 10.1155/2016/4567146

**Published:** 2016-04-27

**Authors:** Chizuru Jinno, Naoki Morimoto, Ran Ito, Michiharu Sakamoto, Shuichi Ogino, Tsuguyoshi Taira, Shigehiko Suzuki

**Affiliations:** ^1^Department of Plastic and Reconstructive Surgery, Graduate School of Medicine, Kyoto University, 54 Kawahara-machi, Syougoin, Sakyou-ku, Kyoto City, Kyoto 606-8507, Japan; ^2^Department of Plastic and Reconstructive Surgery, Kansai Medical University, 2-5-1 Shin-machi, Hirakata City, Osaka 573-1010, Japan; ^3^GUNZE Ltd. QOL Research Center Laboratory, 1 Ishiburo, Inokura Shinmachi, Ayabe, Kyoto 623-8512, Japan

## Abstract

The objective of this study was to compare the effectiveness of the collagen-gelatin sponge (CGS) with that of the collagen sponge (CS) in dermis-like tissue regeneration. CGS, which achieves the sustained release of basic fibroblast growth factor (bFGF), is a promising material in wound healing. In the present study, we evaluated and compared CGSs and conventional CSs. We prepared 8 mm full-thickness skin defects on the backs of rats. Either CGSs or CSs were impregnated with normal saline solution (NSS) or 7 *μ*g/cm^2^ of bFGF solution and implanted into the defects. At 1 and 2 weeks after implantation, tissue specimens were obtained from the rats of each group (*n* = 3, total *n* = 24). The wound area, neoepithelial length, dermis-like tissue area, and the number and area of capillaries were evaluated at 1 and 2 weeks after implantation. There were no significant differences in the CGS without bFGF and CS groups. Significant improvements were observed in the neoepithelial length, the dermis-like tissue area, and the number of newly formed capillaries in the group of rats that received CGSs impregnated with bFGF. The effects on epithelialization, granulation, and vascularization of wound healing demonstrated that, as a scaffold, CGSs are equal or superior to conventional CSs.

## 1. Introduction

We developed a bilayered acellular artificial dermis (Pelnac®, Gunze Co. Ltd., Ayabe, Japan) consisting of an upper silicone sheet and a lower collagen sponge (CS) [1]. After the CS is grafted onto a full-thickness skin defect, fibroblasts and new capillaries spread throughout the lower layer of the sponge. New collagen fibers are synthesized by the penetration of fibroblasts and the collagen sponge biodegrades and are gradually replaced with regenerated dermis-like tissue within a period of 2-3 weeks [1, 2]. Artificial dermis has been used for the treatment of full-thickness skin defects caused by burns, in the waiting period for tumor excision, and for the treatment of intractable ulcers for more than 10 years [3]. However, until the capillaries infiltrate the collagen sponge and the vascular network is formed, the artificial dermis exposes the patient to a high risk of infection [4]. It is therefore difficult to apply artificial dermis to chronic ulcers, such as decubitus, diabetic, and leg ulcers [4]. Basic FGF, which was identified in 1974, promotes the proliferation of fibroblasts and the formation of capillaries and accelerates tissue regeneration [5–9]. In Japan, human recombinant bFGF (Fibrast Spray®, Kaken Pharmaceutical Co., Ltd., Tokyo, Japan) has been available since 2001, and its clinical effectiveness has been verified [7]. In combination with bFGF, artificial dermis has been reported to accelerate dermis-like tissue formation [10]. However, bFGF is rapidly diffused and inactivated after application* in vivo* [11]. To overcome this disadvantage, we developed a CGS that contains a 10 wt% concentration of acidic gelatin which is capable of sustaining positively charged growth factors, such as bFGF, via the formation of ion complexes between bFGF and gelatin [4, 12, 13]. In our previous study, we reported that CGSs impregnated with bFGF (7 *μ*g/cm^2^) accelerated neovascularization and wound healing with less contraction on the backs of mice, in the femoral trochanters of diabetic mice, and in the palatal mucosa of dogs [12, 14]. We also reported that CGSs impregnated with bFGF accelerated the healing process of chronic ulcers in the first-in-human clinical trial [13]. However, the equivalence of CS and CGS as a scaffold has not yet been confirmed. Therefore, in this study, the effects of the application of CSs and CGSs with and without bFGF were evaluated and compared using the same full-thickness skin defects in rats in terms of dermis-like tissue formation, epithelization, and capillary formation.

## 2. Material and Methods

### 2.1. Animals and Operations

The animals were maintained at the Bioscience Department of KAC Co., Ltd. (Rittou, Japan). The number of animals used in this study was kept to a minimum, and all possible efforts were made to reduce their suffering in compliance with the protocols established by the Animal Research Committee Determination of KAC.

### 2.2. Preparation of CS and CGS

We used conventional CSs (Pelnac, Gunze Co. Ltd., Ayabe, Japan) and CGSs produced according to the procedure shown in our previous study [1, 4]. Briefly, a collagen and gelatin solution with a gelatin (isoelectric point (IEP): 5.0) concentration of 10 wt% of the total solute was prepared and freeze-dried. A silicone solution (Shin-Etsu Chemical, Tokyo, Japan) was used to make silicone sheets of 200 *μ*m in thickness containing a polyester mesh, and the top of the CGS was covered with the silicone sheet.

### 2.3. The Impregnation of CS and CGS with NSS and bFGF

We prepared CSs and CGSs of 8 mm in diameter on a dish (NUNC DISH 100X20 VENTS NUNCLON D SI (Thermo Fisher Scientific Inc., Yokohama, Japan)). We previously reported that CGSs impregnated with bFGF at doses of 7 *μ*g/cm^2^ and 14 *μ*g/cm^2^ accelerated the healing processes in mice and diabetic mice, respectively [12]. We therefore adopted the 7 *μ*g/cm^2^ dosage in the present study. At the time of impregnation, 500 *μ*g of human recombinant bFGF, in the form of a dry powder (Fibrast Spray; Kaken Pharmaceutical, Tokyo, Japan), was dissolved in 7.14 mL of normal saline solution (NSS, Otsuka Pharmaceutical Co., Ltd., Tokyo, Japan), and 70 *μ*g/mL (3.5 *μ*g/50 *μ*L) of bFGF solution was prepared. Fifty microliters of NSS was applied to CSs or CGSs in the NSS group; the same amount of bFGF solution was applied to impregnate CSs or CGSs with 7 *μ*g/cm^2^ of bFGF. They were incubated for 30 minutes at room temperature. Four experimental groups were generated as follows: (1) CSs impregnated with NSS (CS-NSS group); (2) CGSs impregnated with NSS (CGS-NSS group); (3) CSs impregnated with bFGF (7 *μ*g/cm^2^) (CS-bFGF group); and (4) CGSs impregnated with bFGF (7 *μ*g/cm^2^) (CGS-bFGF group). Eighteen samples were taken from each experimental group.

### 2.4. The Implantation of CS and CGS into Full-Thickness Skin Defects in Rats

We prepared seven-week-old male rats (*n* = 24, Slc: Wistar, SLC Japan Co., Ltd., Fukuoka, Japan). All of the rats had their backs and abdomens shaved and depilated under anesthesia by the intraperitoneal injection of pentobarbital (30 mg/kg) (Somnopenthyl®, Kyoritsu Seiyaku Corporation, Tokyo, Japan) and the inhalation of isoflurane (Escain® Pfizer Japan Inc., Tokyo, Japan). Three full-thickness skin defects measuring 8 mm in diameter were created in the back (longitudinally) of each rat, and 9 samples from three animals were included in each group. Therefore, each group had 9 samples that were adequate for the statistical analysis. Three full-thickness skin defects were created at an interval of 16 mm using an 8 mm diameter skin punch biopsy tool (Kai Industries, Gifu, Japan), a scalpel, and scissors. The panniculus carnosus was preserved. CSs or CGSs (*n* = 18 in each group) impregnated with either NSS or bFGF were implanted into three skin defects created on the backs of each rat (6 rats in each group). The CSs and CGSs were sutured to the marginal skin using 5-0 nylon sutures (Medical U&A Inc., Osaka, Japan), covered with gauze, and fixed in place with adhesive tape (SILKYTEX®, Alcare Co. Ltd., Tokyo, Japan).

### 2.5. The Assessments of the Wound Area and the Neoepithelial Length

At 1 and 2 weeks after implantation, 3 rats per group were sacrificed via the inhalation of carbon dioxide. After the removal of the silicone sheets, the wounds (*n* = 9) were photographed, and the wound area was measured using the Image J software program (version 1.47, National Institute of Health, USA). The wound area was expressed as the percentage of the original wound area. Skin specimens, including the implanted CSs and CGSs, were harvested using scalpels and scissors and were sectioned axially at the center of each specimen. The specimens were then fixed with 20% formalin fluid (Mildfolm®, Wako Pure Chemical Industries, Osaka, Japan), paraffin-embedded, and sliced into 4 *μ*m thick sections. The sections were then stained with hematoxylin and eosin (H&E). Using a fluorescence microscope (Biorevo BZ-9000; Keyence, Co., Osaka, Japan), the length of the neoepithelium, from the innermost hair root of the marginal skin to the end of the neoepithelium, was measured on each side of each cross-sectional area in each specimen at a magnification of ×100. The sum of the lengths of the epithelium was evaluated on both sides.

### 2.6. Evaluation of the Area of Dermis-Like Tissue

Azan staining sections were used to assess the newly formed dermis-like tissue. Azan stain is a complex staining method for general histology. Nuclei are stained bright red with azocarmine G and connective tissues, such as collagen fibers, are stained blue with aniline blue or orange G. The area of newly formed dermis-like tissue was measured by using the image stitching function of a fluorescence microscope (Biorevo BZ-9000). The lateral border of the regenerated area was determined by the transitional boundary layer of the continuous parallel structure of the dermis and the fragmental structure of newly formed dermis-like tissue. The lower border of the regenerated area was above the panniculus carnosus, and the upper epithelium was not included in the measurement. The area of newly formed dermis-like tissue was statistically analyzed.

### 2.7. Evaluation of Newly Formed Capillaries in the Dermis-Like Tissue

Immunohistological staining with von Willebrand factor antigen was performed to detect newly formed capillaries in the dermis-like tissue. After deparaffinization and rehydration, the sections were incubated in phosphate-buffered saline (PBS Life Technologies Co. Japan, Tokyo, Japan) with 0.1% trypsin (Vector Laboratories Inc., Burlingame, GA, USA) for 15 min at 37°C for antigen retrieval. Anti-von Willebrand factor rabbit polyclonal antibody (1 : 500 dilutions, Dako Japan Co., Tokyo, Japan) and EnVision^+^ Rabbit/HRP (Dako Japan Co., Tokyo, Japan) were used as the secondary antibody. These sections were exposed to DAB (3,3′-diaminobenzidine-4HCL; Dako Japan Co., Tokyo, Japan) for 2 min at room temperature. Counterstaining was performed with hematoxylin.

In each section, the newly formed capillaries were counted and the area of newly formed capillaries was measured manually in the regenerated area at a magnification of ×200, using the measurement module of a fluorescence microscope (Biorevo BZ-9000).

### 2.8. Statistical Analysis

All of the data were expressed as the mean + standard error (SE) and were analyzed using Tukey's significant difference test (Tukey Kramer test). *P* values of <0.05 were considered to indicate statistical significance.

## 3. Results

### 3.1. Wound Area

**Figure 1:**
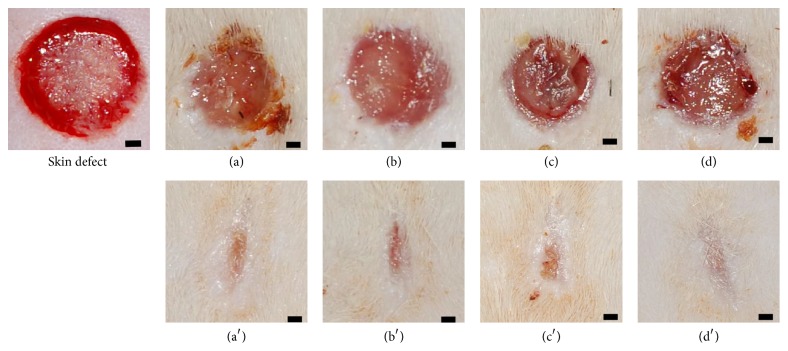
The gross appearance of the full-thickness skin defects before CS and CGS implantation and wounds at 1 and 2 weeks after implantation. The gross appearance of wounds in the CS-NSS (a), CGS-NSS (b), CS-bFGF (c), and CGS-bFGF (d) groups at 1 week after implantation. The gross appearance of wounds in the CS-NSS (a′), CGS-NSS (b′), CS-bFGF (c′), and CGS-bFGF (d′) groups at 2 weeks after implantation. Scale bar: 1000 *μ*m.

**Figure 2:**
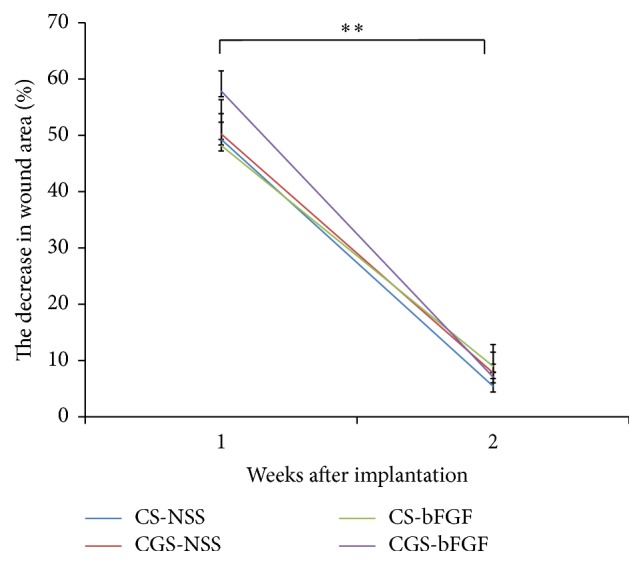
The time course of the remaining wound area. The wounds were treated with CS-NSS, CGS-NSS, CS-bFGF, or CGS-bFGF at 1 and 2 weeks. At 1 and 2 weeks, the remaining wound area did not differ among the groups to a statistically significant extent. At 2 weeks after implantation, the areas of the wounds in all of the groups were significantly smaller than at 1 week (^*∗∗*^ *P* < 0.01).

One animal died suddenly on the fifth day due to unknown reasons. The gross appearance of the wounds at 1 and 2 weeks after implantation is shown in Figure 1. At one week after surgery, wound closure was not completed in any of the four groups. At two weeks after surgery, the wound area was reduced and almost completely epithelized, and there were no signs of pus, redness, or inflammation in any of the four groups. The time course of the wound area is shown in Figure 2. At two weeks after implantation, the wound area in each group was significantly smaller than at 1 week (*P* < 0.01). There were no significant differences in wound areas of the experimental groups at 1 and 2 weeks after surgery (Figure 2).

### 3.2. Histological Assessment of the Neoepithelial Length

**Figure 3:**
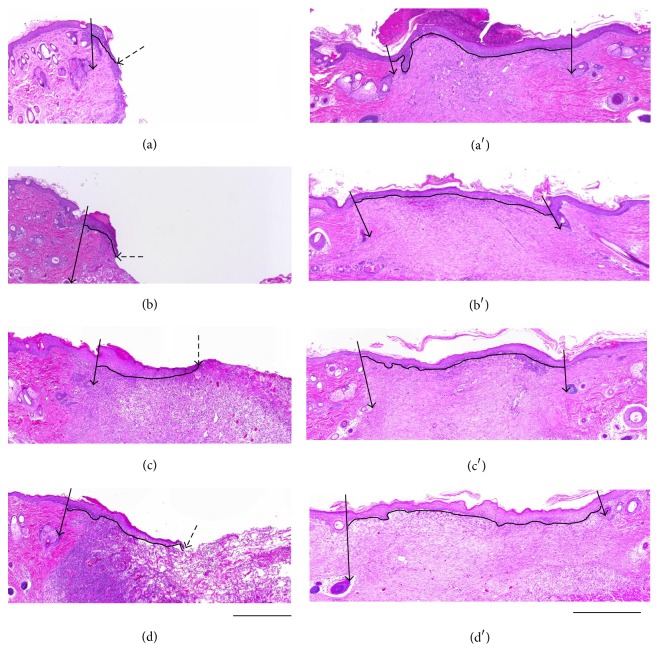
Light micrographs of full-thickness skin defects at 1 and 2 weeks after implantation. The wounds of the CS-NSS (a), CGS-NSS (b), CS-bFGF (c), and CGS-bFGF (d) groups at 1 week. The wounds of the CS-NSS (a′), CGS-NSS (b′), CS-bFGF (c′), and CGS-bFGF (d′) groups at 2 weeks. The black arrow with a solid line indicates a hair root which is the edge of the regenerated dermis-like tissue. The black arrow with a broken line indicates the end of the neoepithelium. The neoepithelium is shown in the upper section as a black line. Scale bar: 500 *μ*m.

**Figure 4:**
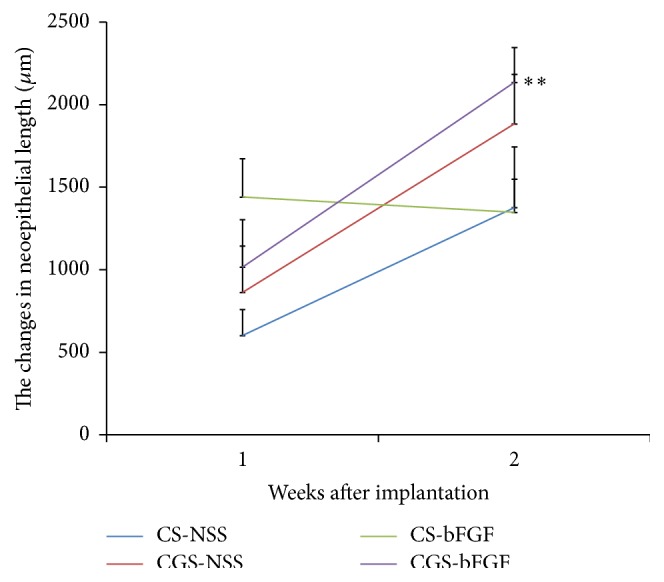
The time course of the neoepithelial length. The wounds of the CS-NSS, CGS-NSS, CS-bFGF, and CGS-bFGF groups at 1 and 2 weeks. At 2 weeks, the neoepithelial length of the CGS-bFGF group was significantly longer than that of the CS-NSS and CGS-NSS groups at 1 week (*P* < 0.01 and *P* < 0.05, resp.). At 2 weeks, the neoepithelial length of the CGS-NSS group was significantly longer than that of the CS-NSS group at 1 week (*P* < 0.05). At 2 weeks after implantation, the neoepithelial length of the CGS-bFGF group was significantly longer than at 1 week (^*∗∗*^ *P* < 0.01).

Light microphotographs of the histological sections (H&E staining) at 1 and 2 weeks after implantation are shown in Figure 3. The time course of the neoepithelial length is shown in Figure 4. At two weeks, the neoepithelial length of the CGS-bFGF group was significantly longer than that of the CS-NSS and CGS-NSS groups at 1 week (*P* < 0.01 and *P* < 0.05, resp.). At two weeks, the neoepithelial length of the CGS-NSS group was significantly longer than that of the CS-NSS group at 1 week (*P* < 0.05). At two weeks after implantation, the neoepithelial length of the CGS-bFGF group was significantly longer than at 1 week (*P* < 0.01) (Figure 4).

### 3.3. Evaluation of the Area of Dermis-Like Tissue

**Figure 5:**
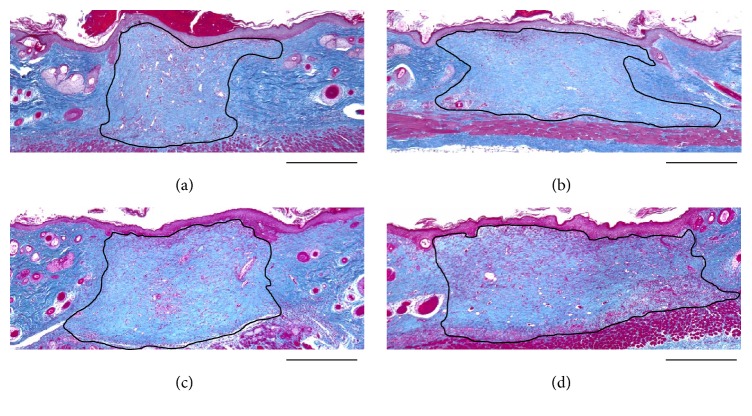
Light micrographs (Azan staining) of the wounds at 2 weeks after implantation. The wounds of the CS-NSS (a), CGS-NSS (b), CS-bFGF (c), and CGS-bFGF (d) groups. The area surrounded with black line shows newly formed dermis-like tissue. Scale bar: 500 *μ*m.

**Figure 6:**
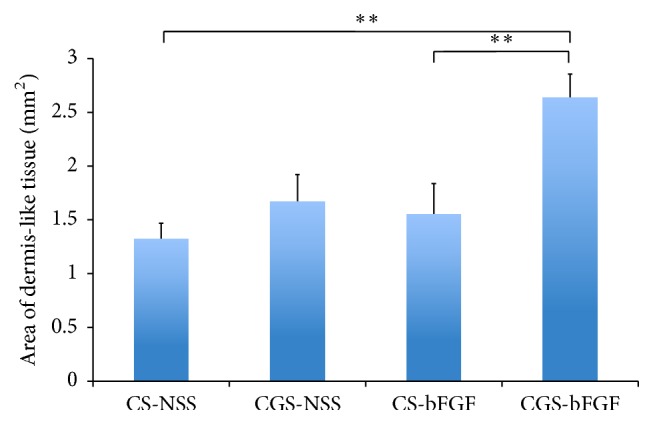
The comparison of the area of newly formed dermis-like tissue at 2 weeks. The newly formed dermis-like tissue in the wounds of the CGS-bFGF group was significantly larger than that of the CS-NSS and CS-bFGF groups at 2 weeks (^*∗∗*^ *P* < 0.01).

The regenerated area of dermis-like tissue at two weeks after implantation is shown in Figure 5. The area of dermis-like tissue in the CGS-bFGF group was significantly larger than that in the CS-NSS and CS-bFGF groups (*P* < 0.01) (Figure 6).

### 3.4. Evaluation of Newly Formed Capillaries in the Dermis-Like Tissue

**Figure 7:**
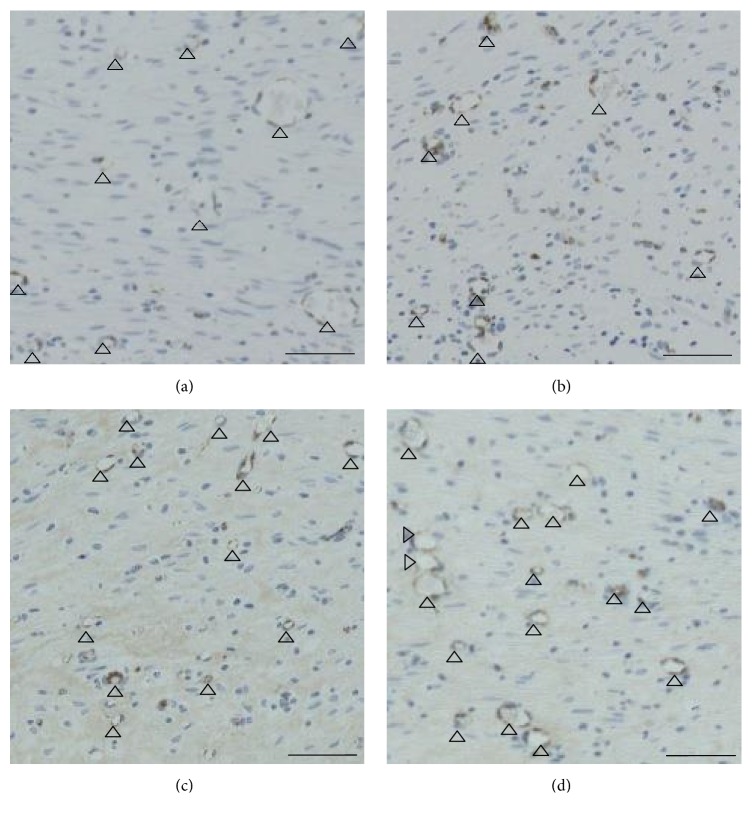
Immunohistochemical staining (von Willebrand factor antibody) of newly formed capillaries at 2 weeks after implantation. The wounds of the CS-NSS (a), CGS-NSS (b), CS-bFGF (c), and CGS-bFGF (d) groups. The open arrowheads indicate newly formed capillaries. Scale bar: 50 *μ*m.

**Figure 8:**
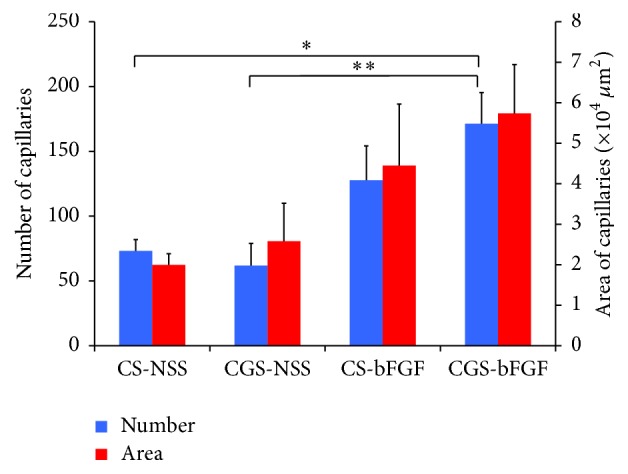
The comparison of the number and area of newly formed capillaries at 2 weeks after implantation. The number of newly formed capillaries in the wounds treated of the CGS-bFGF group was significantly higher than that of the CS-NSS and CGS-NSS groups (^*∗*^ *P* < 0.05 and ^*∗∗*^ *P* < 0.01, resp.).

Light micrographs (von Willebrand factor antibody staining), which were captured at 2 weeks after surgery, are shown in Figure 7. The images show the center area of dermis-like tissue specimens of 250 *μ*m in width and height. The number of newly formed capillaries in the CGS-bFGF group was significantly larger than that in the CS-NSS and CGS-NSS groups (*P* < 0.05 and *P* < 0.01, resp.) (Figure 8).

## 4. Discussion

In the present study we showed the efficacy of CGSs impregnated with bFGF in the acceleration of the wound healing process. We applied conventional CSs and CGSs (a novel scaffold that allows for the sustained release of growth factors) with and without bFGF to skin defects in rats and compared the wound healing process. A significantly higher number of capillaries were observed in the CGS-bFGF group; however, the area of newly formed capillaries was not significantly higher. This is probably because the number of specimens in the CS-NSS group was small due to the unexpected death of an animal. In other respects, CGSs impregnated with bFGF (7 *μ*g/cm^2^) accelerated angiogenesis and dermis-like tissue formation. This finding is in line with the findings of our previous studies using mice, diabetic mice, rabbits, and beagle dogs [4, 12, 14]. We emphasize the formation of dermis-like tissue in the healing process, because it accelerates epithelization and prevents wound contracture [14]. Two mechanisms are involved in the wound healing process: wound contraction and epithelization. Murine wounds close mostly through wound contraction. In humans, wounds close mostly through epithelization and wound contraction causes scar contracture, decreasing the range of joint motion and/or causing hypertrophic scarring. The CGS-bFGF group was the only group in which the neoepithelial length was observed to grow from the first week to the second week. This growth was the result of dermis-like tissue formation (Figure 4). Conventional CSs have been used for the preparation of dermis-like tissue, which prevent scar contracture. In this point, the CGS-bFGF group showed the highest level of dermis-like tissue formation, which indicates that CGSs impregnated with bFGF can prevent wound contracture. In our previous studies, we developed CGSs impregnated with bFGF mainly to accelerate the healing of chronic ulcers (such as diabetic foot ulcers and venous leg ulcers); however, the present results indicate that, in addition to conventional CSs, CGSs impregnated with bFGF can be used to promote dermis-like tissue formation in the treatment skin defects.

CGS contains 10 wt% of gelatin, which degrades more easily than collagen. Another concern with regard to CGSs is the possibility that its earlier degradation (in comparison to CSs) after application* in vivo* is not a desirable characteristic for a scaffold. However, we did not observe a significant difference in the area of dermis-like tissue formation in the CS-NSS and CGS-NSS groups and the CGS-bFGF group showed the largest area of dermis-like tissue. These results indicate that CGSs are equal to CSs in their efficacy as scaffolds and that CGSs were not easily degraded in the accelerated angiogenesis that occurred as a result of their impregnation with bFGF.

Recently, skin substitutes containing living cells have been reported to be effective in the treatment of chronic skin ulcers [15–18]. We have reported that CSs cultured with autologous fibroblasts and CGSs impregnated with bFGF were equally effective in the treatment of diabetic ulcers [19]. We have not evaluated the combination of CGSs with living cells; however, it is thus expected that CGSs could be used in combination with cells as well as CSs. Various types of cells, including stem cells, have been used in the treatment of skin ulcers [20–22]. CGSs can achieve the sustained delivery of positively charged growth factors and can also be used as a cell carrier. In the future, other growth factors and cells will be developed. It is possible that these growth factors and cells will be combined with our CGS and used for treatment; thus, our CGS has the potential to be used in the coming era of regenerative medicine.

## 5. Conclusions

Wound healing after the treatment of rodent skin defects with conventional CSs and CGSs with or without bFGF was compared. The results showed that, as a scaffold, CGSs can equal the efficacy of conventional CSs without bFGF. Furthermore, we found that CGSs impregnated with bFGF (7 *μ*g/cm^2^) accelerated the wound healing process which indicates that they may supersede conventional CSs in the clinical setting.
